# A dataset on two decades of Japan's energy investments in Southeast Asian countries, 2000–2020

**DOI:** 10.1016/j.dib.2023.109818

**Published:** 2023-11-20

**Authors:** Laurence L. Delina, Chloe Chan, Timothy Joseph Henares

**Affiliations:** Division of Environment and Sustainability, The Hong Kong University of Science and Technology, Clear Water Bay, Kowloon, Hong Kong

**Keywords:** Japan, Southeast Asia, Energy sector, Energy development, Energy finance, Energy funding, Renewable energy finance

## Abstract

Between 2000 and 2020, Japan played a crucial role in transforming Southeast Asia's energy sector, contributing significantly to local, national, and regional development. This period saw substantial Japanese investments in various energy sources, including renewable energy initiatives and continued use of fossil fuels, while emphasising capacity development. Our research involved an extensive and systematic inventory of secondary resources, with meticulous validation and fact-checking of critical project data. The resulting dataset provides funding amounts and details channelled through Japanese government-backed institutions and private corporations. This dataset could enhance our understanding of Japan-supported energy infrastructure and soft-skill capacity-building projects, allowing us to analyse further how these investments were aligned with the broader economic and sustainability objectives of Southeast Asian countries. Covering investment types, funding sources, and project locations, this valuable resource is useful for scholars across disciplines. Asian studies researchers can gain insights into Japan's strategic involvement in the region, while energy studies specialists can gain a nuanced understanding of the evolving energy finance landscape. Energy policy experts can also use this data to analyse the implications of Japan's contributions to Southeast Asia's energy transition.

Specifications Table

**Table:** 

Subject	Social Sciences / Geography
Specific subject area	An inventory of Japan's energy-related investments in Southeast Asian countries from 2000 to 2020
Data format	.xlsx file (Raw, analysed, and filtered)
Type of data	Tables, charts, graphs, figures
Data collection	We conducted an extensive and systematic inventory of secondary resources, meticulously validating and fact-checking the information. This data collection and fact-checking process spanned from May 24, 2021 to September 12, 2022.
Data source location	The data were gathered from various sources, including publicly accessible government and private sector/corporate websites and news accounts. Each data point includes corresponding references. The raw data is securely stored in the databases of The Hong Kong University of Science and Technology and at Harvard Dataverse. These primary sources are listed in the dataset, particularly in the last columns of the sheet named “Dataset_JPinvestmentSEA.”
Data accessibility	Repository name: Harvard Dataverse All data can be accessed at [1]: https://doi.org/10.7910/DVN/6N1EYH

## 1 Value of the Data

• This dataset provides a holistic view of Japan's investments in the energy sector of Southeast Asia from 2000 to 2020. It serves as a valuable resource illuminating the intricacies of the region's energy landscape.
• One of the key strengths of this dataset is its robust data collection methodology. It draws from diverse sources, including both governmental and private sector websites. This multi-sourced approach not only enhances the reliability and accuracy of the dataset but also bolsters credibility, making it an indispensable tool for researchers.
• A distinguishing feature of this dataset is the meticulous referencing of each of its 372 data points. This meticulous referencing ensures the dataset's credibility and facilitates rigorous academic and policy analysis while maintaining transparency.
• The dataset presents different categories (e.g., Fossil Energy, Renewable Energy, etc.) in which the user can split and analyse the data for in-depth exploration and comparison of Japan's investments in Southeast Asia's energy sector, thereby enabling more nuanced and focused investigations within these specific energy domains.
• This dataset offers versatility in its application. Researchers can perform various statistical analyses using descriptive statistics, regression, cluster, and factor analyses.
• Scholars across different academic disciplines can leverage this dataset for their research. Geography, energy studies, Asian studies, energy policy, and international relations are amongst the fields that can benefit from this dataset. Using this resource, researchers can gain a nuanced understanding of Japan's role in Southeast Asia's energy development. This, in turn, fosters cross-disciplinary research and insights.

## 2 Data Description

The dataset contains information on 372 energy development-related projects supported by Japan across ten Southeast Asian countries and subregions from 2000 to 2020 [1]. The data is organised into four sheets within a single Excel file, and an overview is provided in Table 1. Japan's support during this period extended to various locations within the Southeast Asian region, including Brunei Darussalam (1 project), Cambodia (34 projects), Indonesia (69 projects), Lao PDR (21 projects), Malaysia (18 projects), Myanmar (28 projects), the Philippines (48 projects), Singapore (8 projects), Thailand (35 projects), Vietnam (876 projects), and the broader regional context (4 projects).

**Table 1:** Overview of the dataset and sheets (DOI:10.7910/DVN/6N1EYH).

Dataset	Sheet	Data in sheet
Inventory of Japan-funded energy development-related projects in Southeast Asian countries, 2000–2020	Dataset_JPinvestmentSEA	Recipient countries; Project titles as in primary source; Capacities of installed project; Funding amount; Disbursement years; Typologies; Project start and end-dates; Consortia; References
Currency conversion and inflation tables, 2000–2020	CurrencyConv-and-InflationTable	Currency conversion and inflation tables
General summaries in charts and graphs of the inventory	consolidated_graphsandfigures	Charts and figures
Country-specific summaries in charts and graphs of the inventory	country_figures	Charts and figures

These projects are classified into one or more of the five typologies delineated in Delina [2] (1): fossil energy (2), sustainable energy (3), large hydropower (4), electricity transmission and distribution, and (5) policy, reform, capacity development, and planning support. Furthermore, the projects have various statuses as of December 31, 2020, including (1): approved for funding (2), cancelled (3), completed (4), completed with remaining works (5), incomplete (6), MOU signed (7), ongoing (8), planning, and (9) unknown.

The sheet labelled “Dataset_JPinvestmentSEA” contains information on the recipient country for each of the 372 Japan-supported energy-related projects. Each project is allocated one or more rows to facilitate inflation adjustments. The variables include the project capacity (in MW or other units), location, borrower or recipient of funding, total funding amount in USD as announced, total funding amount in USD adjusted for inflation using data from [[3], [4], [5], [6], [7], [8]], project typology following Delina [2], project start and end dates, project status, and project partners and contractors. Blank cells or cells containing ‘N/A’ indicate data unavailability.

The “CurrencyConv-and-InflationTable” sheet provides details of currency conversion and the US Consumer Price Index from 2000 to 2020. It is essential to note that rates highlighted in blue cells are for computations, while rates in red cells represent figures calculated from existing data sources, all duly referenced within the sheet.

For numerical data and corresponding visualisations, refer to the sheet titled “consolidated_graphsandfigures.” This sheet features block cells on the left, with distinct dark and light yellow colours. Each block is accompanied by a brief description, presented in bold text. The numbers enclosed in brackets denote the specific figures derived from these tables.

The data in the dark yellow blocks remains unaltered and contains cross-references to the sheet titled “Dataset_JPinvestmentSEA.” Conversely, the light yellow blocks have undergone further modification, with numerical values truncated to millions, where feasible. The dataset also includes eight sets of figures, with Figs. 1 to 6 using data from the light yellow blocks, while Figs. 7 and 8 draw from the dark yellow blocks.

1. Fig. 1 shows the percentage investment, measured by monetary value, within each of the five project typologies for each country and region. This data is visually represented through a stacked bar chart, a percentage bar chart, and a bar chart. Additionally, Fig. 1.1 offers an overall depiction of the percentage distribution of each investment type, calculated by monetary value, using pie and radar charts.
2. Fig. 2 presents the monetary value invested in each country and region from 2000 to 2020, adjusted to USD and expressed in millions for 2020. The visualisations include stacked bar charts, stacked line charts, and line charts. Furthermore, Fig. 2.1 uses line and bar charts to illustrate Japan's total monetary investment from 2000 to 2020, adjusted to USD for 2020.
3. Fig. 3 outlines the total amount invested by Japan in each country, denominated in millions of USD for the year 2020. Visual representations include bar charts, percentage pie charts, and treemaps.
4. Fig. 4 details the total number of projects categorised by project typology from 2000 to 2020 within each country and region. This information is visually conveyed through radar charts, percentage radar charts, and stacked bar charts.
5. Fig. 5.1 offers insight into the total number of projects within each project typology, showcasing trends from pre-2000 to 2020. The visualisations include stacked line charts, stacked bar charts, and percentage bar charts. Fig. 5.2 represents the same data using a percentage pie chart, while Fig. 5.3 simplifies the information by grouping years into five-year intervals using similar chart formats.
6. Fig. 6 delineates the monetary value invested by the top 5 Japanese lenders across all available projects, categorised by project typology and adjusted to USD million values for 2020. The visualisations include radar charts, percentage bar charts, and bar charts.
7. Fig. 7 offers insights into the total capacity in MW invested across each country and region, depicting trends from 2000 to 2020. Visualisations comprise area charts, bar charts, and stacked bar charts.
8. Fig. 8 focuses on the total capacity in MW invested per project type, featuring trends from 2000 to 2020. This analysis includes three of the five project typologies listed in [2]. The visualisations comprise stacked bar charts, area charts, and radar charts.

To access additional visual representations of data for individual countries and the Southeast Asian region, one can refer to the “country_figures” sheet, which includes Figs. 1, 2, and 8. These figures offer unique data presentations. Figs. 1 and 2 display the total monetary value invested in each project typology and country, adjusted to USD million for 2020, using pie charts, radar charts, line charts, and bar charts. Fig. 8 provides information on invested capacity in MW within each project typology and country, featuring trends from 2000 to 2020 through stacked bar charts, area charts, and radar charts.

## 3 Experimental Design, Materials and Methods

The dataset contains raw and filtered data collected, verified, and fact-checked between May 24, 2021 and September 12, 2022. The data collection process involved a systematic review of various sources, including Japan-funded institution websites, documents, and databases such as the Japan Bank for International Cooperation, Japan International Cooperation Agency, the World Bank, the Asian Development Bank, and embassy websites of Japanese and Southeast Asian countries. The period of inquiry spanned from 1950 to 2020. Delina provided guidelines on which data to collect, and Gonzaga established the preliminary database from May 2021 to May 2022. A three-member team, led by Chan, cross-checked and verified the details for each project listed in the database, analysed the dataset, and generated the figures and tables from May to September 2022, with regular feedback and cross-checking from Delina.

To ensure completeness, a triangulation method was systematically applied to encompass a broad spectrum of projects, incorporating most, if not all, relevant projects. The data examination was extended back to 1950 to account for projects initiated before the year 2000 that were either ongoing or had concluded within the timeframe of this study.

Data collection, verification, and fact-checking involved cross-referencing project titles (under “Project description” in column C) with existing reports, documents, or news articles. Information, such as project capacity, project loan amount, disbursement period, duration of the loan, and consortium details (refer to column headings in the sheet titled “Dataset_JPinvestmentSEA” for the complete list), were extracted from these documents and recorded in the database. Disbursement data was assumed to be disbursed each year over the years of disbursement.

In addition to the core data collection and fact-checking efforts, inflation, currency conversion, and US Consumer Price Index information were collected from June to July 2022 using data from [[3], [4], [5], [6], [7], [8]]. Key variables of interest, such as amount invested, top lenders, investment per project type, and total number of projects, were extracted from the “Dataset_JPinvestmentSEA” sheet and organised into tables within the “consolidated_graphsandfigures” sheet. These variables were used to create eight sets of figures, which were generated using Excel's Charts function. For specific references to the websites and documents consulted during this data compilation process, please refer to the references section within the dataset.

## Limitations

The dataset has significant limitations affecting the completeness of the data. Firstly, we could not claim comprehensiveness since not all data will ever be complete due to the extensive variables within each project and the numerous projects considered. Secondly, the dataset may have missed projects that were cancelled, altered, or postponed after December 31, 2020 but have not been reported publicly. Thirdly, some primary sources were translated using Google Translate, which may lead to minor inaccuracies. Fourthly, the absence of precise information on project location, funding sources, contractors, and loan-versus-grant distinctions suggests potential gaps. For example, the funding amount at the announcement of the project, capacity to be installed by the project, and number of years this funding was disbursed over could be missing. Also, incorrect names, dates, and hydropower plant spellings made cross-checking challenging, indicating potential issues in data verification and validation.

## Ethics Statement

The current work does not involve human subjects, animal experiments, or any data collected from social media platforms. We did not need permission for using the primary data mentioned in the Data Specifications table.

## CRediT authorship contribution statement

**Laurence L. Delina:** Conceptualization, Methodology, Resources, Data curation, Writing – original draft, Writing – review & editing, Supervision, Project administration, Funding acquisition. **Chloe Chan:** Methodology, Validation, Formal analysis, Data curation, Investigation, Writing – original draft, Writing – review & editing, Visualization. **Timothy Joseph Henares:** Methodology, Data curation, Writing – original draft, Writing – review & editing.

## Data Availability

Energy-related Japanese investments in Southeast Asia, 2000 to 2020 (Original data) (Dataverse)
